# A perturbative approach to study information communication in brain networks

**DOI:** 10.1162/netn_a_00260

**Published:** 2022-10-01

**Authors:** Varun Madan Mohan, Arpan Banerjee

**Affiliations:** National Brain Research Centre, Manesar, India

**Keywords:** Information flow, Perturbation, Network-dynamical interactions, Computational modeling, Response asymmetry

## Abstract

How communication among neuronal ensembles shapes functional brain dynamics is a question of fundamental importance to neuroscience. Communication in the brain can be viewed as a product of the interaction of node activities with the structural network over which these activities flow. The study of these interactions is, however, restricted by the difficulties in describing the complex dynamics of the brain. There is thus a need to develop methods to study these network-dynamical interactions and how they impact information flow, without having to ascertain dynamics a priori or resort to restrictive analytical approaches. Here, we adapt a recently established network analysis method based on perturbations, it to a neuroscientific setting to study how information flow in the brain can raise from properties of underlying structure. For proof-of-concept, we apply the approach on in silico whole-brain models. We expound on the functional implications of the distributions of metrics that capture network-dynamical interactions, termed *net influence* and *flow*. We also study the network-dynamical interactions at the level of resting-state networks. An attractive feature of this method is its simplicity, which allows a direct translation to an experimental or clinical setting, such as for identifying targets for stimulation studies or therapeutic interventions.

## Author Summary

In this work, we use controlled perturbations in silico to identify regions that influence and mediate information flow in active brain networks. Conventional approaches of identifying such regions require the extensive analytical treatment of mathematical models describing node dynamics, thus restricting its scope only to systems where such models have been defined. The presented formalism can identify regions of dynamical and functional importance by simply measuring responses to perturbations, and can thus be applied at any scale where regions can be perturbed, and without any prerequisite information about node dynamics. Furthermore, the relation of metrics to interregional communication, functional capabilities, and structure-function mapping in general affords them considerable practical importance, especially for identifying targets for therapeutic interventions.

## INTRODUCTION

The brain is a complex biological network in which healthy function strongly relies on the efficient transmission of information between functional entities at multiple scales (Avena-Koenigsberger, Misic, & Sporns, 2018; Battaglia, Benchenane, Sirota, Pennartz, & Wiener, 2011; Goñi et al., 2014). At the whole-brain scale, it is generally assumed that information *flows* along channels of white matter tracts connecting gray matter regions, subsequently leading to the observed brain dynamics. However, determining the precise mapping between the structural connectome (SC), and the functional connectivity (FC) arising from the observed brain dynamics is an enduring problem in neuroscience (Honey, Thivierge, & Sporns, 2010; Suárez, Markello, Betzel, & Misic, 2020). The crucial link to this mapping lies in the particulars of dynamics that evolve over the SC (Forrester, Crofts, Sotiropoulos, Coombes, & O’Dea, 2020). The dynamical complexity of the brain is well showcased by the mathematically diverse models used to describe function at multiple spatial scales (Deco et al., 2013; FitzHugh, 1961; Hodgkin & Huxley, 1952; Naskar, Vattikonda, Deco, Roy, & Banerjee, 2021; Stefanescu & Jirsa, 2008; Wilson & Cowan, 1972). In addition to the complexity introduced at the node level by these models, an added layer of complexity in brain function arises from the combination or interaction of such dynamics with the brain’s underlying structural network. These *network-dynamical interactions* have previously been implicated in the rise of emergent phenomena and functional asymmetries that transcend understanding from local dynamics or structural properties alone (Dayan & Abbott, 2001; Gu et al., 2015; Hansen, Battaglia, Spiegler, Deco, & Jirsa, 2015; Hens, Harush, Haber, Cohen, & Barzel, 2019; Mišić et al., 2018; Moon, Lee, Blain-Moraes, & Mashour, 2015; Novelli, Atay, Jost, & Lizier, 2020; Seguin, Razi, & Zalesky, 2019; Seguin et al., 2019; Sporns, 2014; Strogatz, 2000). However, obtaining insights into how network-dynamical interactions result in patterns of information flow and other dynamical processes is seldom straightforward, often requiring one to resort to analytical approaches. Such an approach, however, requires that there be a well-defined mathematical model for the dynamics to begin with, which limits our understanding to a select few scenarios. Therefore, there is a need for methods that can bypass these conventional prerequisites to understanding these interactions that underlie interregional communication, functional capabilities of brain regions, and structure-function mapping in general. Recent advances in studies of real-world networks (Harush & Barzel, 2017; Hens et al., 2019) have proposed a theoretical framework to tackle problems of this nature.

**Figure 1.**
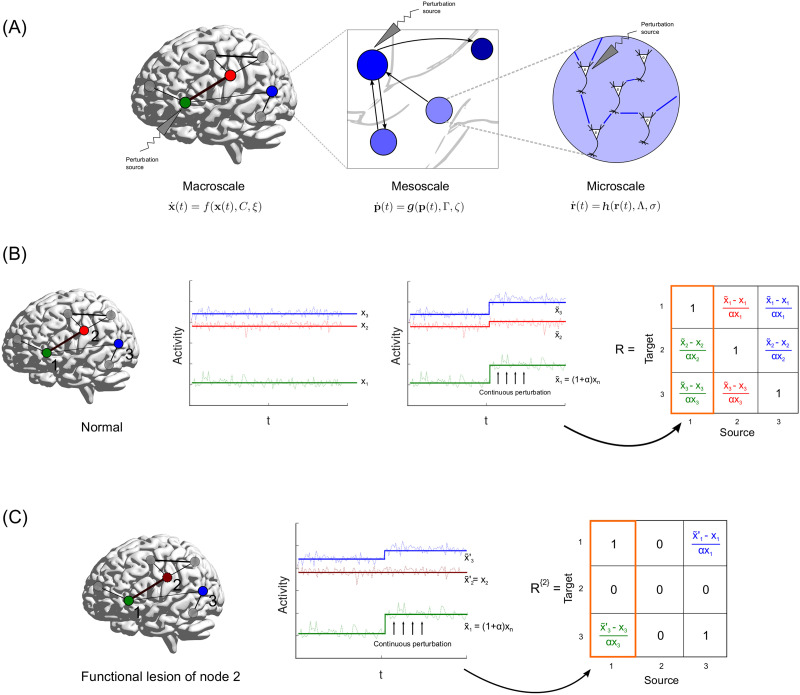
Schematic of perturbation protocol and response matrix before and after freezing node activity. (A) The multiscale scope of the perturbative formalism. Each scale has its own dynamical model evolving over associated networks *C*, Γ, and Λ. Perturbing the functional units results in changes in activity across the rest of the connected network, in accordance with the dynamics in that scale. This is then translated to a region’s influence and role in communication. (B) Nodes 1 (Green), 2 (Red), and 3 (Blue) are active in a whole-brain network. The steady-state values at which they stabilize are given by *x*_1_, *x*_2_, and *x*_3_, respectively. A continuous perturbation of node 1, by an amount *α* results in the activities of node 2 and 3 to stabilize at new steady-state values, $\tilde{x}$_2_ and $\tilde{x}$_3_, respectively. This is used to populate the first column of the linear response matrix (highlighted in orange). The colors of the entries of the response matrix *R*, correspond to the response calculated for a perturbation of the node associated with the color. (C) In order to gauge the contribution of node 2 in eliciting the responses of the other nodes, we perform a functional lesion (freeze the activity of node 2 at its original steady state). The associated columns and rows of node two are thus 0, indicating that node 2 does not respond to any perturbations. Note that this lesion of 2 leads to a different response of 3 to the perturbation of 1, with a new steady state at ${\tilde{x}}_{3}^{\prime }$.

The fallouts of interactions between the dynamics and the underlying structural network can be studied by tracing the effects of changes to a node’s activity (akin to the information) on the rest of the active network—a perturbative approach. Perturbative methods have been previously used in silico to gather insights into diverse aspects of brain function (Deco et al., 2018; Gollo, Roberts, & Cocchi, 2017; Sanz Perl et al., 2021; Veit et al., 2021). Here, we choose to recast the information flow formalism introduced recently in network science (Harush & Barzel, 2017). The transfer of information can be captured as baseline activity perturbation that originates in one region and results in a chain of perturbations in subsequent neighborhoods of intermediary nodes till it reaches the target (Figure 1B). We observe that perturbations can be used to unearth asymmetries in the network influencing capabilities of distinct brain regions, quantified by a metric termed *net influence*. Furthermore, an extension of this formalism leads to the definition of a dynamics-dependent communication centrality measure, termed *flow*, which quantifies the overall contribution of a region to information transfer events. This metric can hold considerable insights into possible communication patterns supported by neurodynamical models. The prime advantage of the perturbative approach is that the method does not alter the structure of the underlying anatomical network. This preserves structural network properties and helps in singling out dynamical consequences to the perturbations. Moreover, the simplicity and mathematical basis of this formalism permits its easy application onto any experimental framework using perturbations, such as brain stimulation techniques. Most importantly, since the measures developed are at the level of time series obtained at any voxel or sensor, the approach is suitable for studies of single neurons to macroscopic brain recordings and is essentially scale-invariant (Figure 1A).

In order to demonstrate the method, we resort to an in silico approach at the whole-brain scale, using an empirically derived SC that supports established whole-brain node dynamics. This is done merely as a proof of concept, and the formalism is not restricted to such approaches alone. First, we apply the perturbation protocol on the mean field model (MFM) (Deco et al., 2013) and look at response asymmetries (net influence distributions) that arise at the level of single brain regions (Figure 1B). We then implement a functional lesion approach to compute the metric Flow, which quantifies the centrality of regions in mediating information flow (Figure 1C). We also show that the distributions of the metrics for MFM dynamics are qualitatively similar over two largely different datasets. We then compute the average net influence and flow of nodes classified in terms of their association to resting-state networks (RSNs), to gauge the typical dynamical influence and information flow mediation capabilities of the RSNs. We also carry out the perturbation protocol on the linear stochastic model (LSM; Galán, 2008) and compare the results with that of the MFM.

We introduce the perturbation protocols and associated metrics in the Methods section. Results for the in silico MFM over the multimodal imaging and connectome analysis–microstructure-informed connectomics (MICA-MICs) dataset is presented in the Results section, and results from the alternate Cambridge Centre for Ageing and Neuroscience (Cam-CAN) dataset and LSM is shown in the Supporting Information figures. In the final section, we discuss the plausibility, functional implications, and limitations of our findings.

## METHODS

### Net Influence and Flow

We introduce two concepts: (1) net influence of a brain subnetwork and (2) the notion of “flow,” which help illuminate and quantify network-dynamic interactions. We implement the method developed in Harush and Barzel (2017) on whole brain neurodynamical models, whose topological architecture are constrained by empirically derived structural brain networks. In an active communicating network, information or activity between two nonneighboring nodes is relayed by intermediary nodes. The original activity or a perturbation of the source node elicits a perturbation in the activity of its immediate neighbors, which then elicit responses in their neighbors and so on, till the effect of the source perturbation finally reaches the target. This perturbation transmission can be easily traced in silico, and the final effect of perturbing the source can be quantified by computing the change in activity of the target per unit change of source activity (Figure 1B).

In general, any neurodynamical model can be represented as

$$\dot{x}\left(t\right)=f\left(x\left(t\right),C,\xi \right)$$ (1)

where **x**(*t*) is the vector of time-dependent activities of every region, *C* represents the network on which the dynamics evolves, and *ξ* is the noise function. If the system is stable, permitting it to evolve for long enough without any external perturbation drives it to its steady state. We can introduce a continuous perturbation held at a constant strength to a region *n*, which has the effect of driving the system to a new steady state. The steady state linear response matrix of a region *m* upon a small perturbation of a region *n*, such that the perturbed state of the source, $\tilde{x_{n}}$ = (1 + *α*)*x*_*n*_ (where *x*_*n*_ denotes the steady state activity of region *n*), is computed as

$$R_{mn}=\left|\frac{\frac{{dx}_{m}}{x_{m}}}{\frac{{dx}_{n}}{x_{n}}}\right|=\left|\frac{\frac{\tilde{x_{m}}-x_{m}}{x_{m}}}{\frac{\tilde{x_{n}}-x_{n}}{x_{n}}}\right|=\left|\frac{\tilde{x_{m}}-x_{m}}{\alpha x_{m}}\right|$$ (2)

where *x*_*m*_ is the steady state of node *m* in the unperturbed system, $\tilde{x_{m}}$ is the new steady state of *m* after perturbation, and *α* quantifies the amount of perturbation. We implement this computationally by solving the equations of all regions *m*, while maintaining the activity of region *n* at (1 + *α*)*x*_*n*_. Computing all pairwise responses in this manner generates the linear response matrix, *R*. The total response elicited by a region *n* is then given by

$$Z_{n}=\sum_{\begin{array}{c} m=1\\ m\neq n \end{array}}^{N}R_{mn}$$ (3)

We define the net influence of a putative brain region *i* to be

$$I_{i}=\sum_{m=1}^{N}R_{mi}-\sum_{m=1}^{N}R_{im}.$$ (4)

Thus, net influence essentially captures the response asymmetry of a node by taking the difference between magnitude of response elicited *on* the rest of the network (sum over rows) and magnitude of response effected *by* the rest of the network (sum over columns). A region that is capable of influencing the rest of the network more than being influenced *by* the network will have a positive value of net influence—termed “influencers.” On the other hand, regions that are instead easily influenced by the network but unable to change the dynamics of the rest of the network to the same extent will have negative net influence—termed “followers.”

Probing the effect of a region *i* on the response of *m* elicited by the perturbation of *n* requires carrying out the procedure mentioned above, but now with the activity of *i* fixed at its unperturbed steady-state value *x*_*i*_ (Figure 1C): an effective “functional lesion.” This is tantamount to *i* remaining at its stable baseline, not being perturbed by *n*, and thereby removing its contribution to $\tilde{x_{m}}$. Computing the response of all possible target source pairs by freezing the activity of *i* generates *R*^{*i*}^. However, computing *R*^{*i*}^ for all *i* is a computationally intensive process, and so we implement an approximation as used in Harush and Barzel (2017), which holds true only for small perturbations, and only requires *R*:

$$R_{mn}^{\left\{i\right\}}\approx R_{mn}-R_{mi}R_{\text{in}}$$ (5)

The difference between *R*^{*i*}^ and *R* is captured by the measure *flow* through region *i*, computed in two steps, as follows:

$$F_{n}^{i}=\frac{Z_{n}-Z_{n}^{\left\{i\right\}}}{Z_{n}}$$ (6)

where $F_{n}^{i}$ is the flow through node *i* for a perturbation source at *n*, to all possible targets, followed by:

$$F^{i}=\frac{1}{N}\sum_{n=1}^{N}F_{n}^{i}$$ (7)

where $Z_{n}^{\left\{i\right\}}$ is the total response elicited by perturbing *n*, when the activity of *i* is frozen, and *F*^*i*^ is the flow averaged over all possible sources, resulting in the effective consideration of all pairwise information transfer scenarios through the node *i*.

A region with a high value of flow would imply its central role in arbitrary information transfer processes in the brain. Since we consider all possible pairwise information transfer scenarios, of which valid communication pathways will form a subset, regions with high flow can be viewed as being central to communication in the network.

It must be noted that both the net influence and flow are measured from the response matrix, whose values are essentially steady-state deviations in response to perturbations. The response matrix, and any metrics derived from it will thus naturally depend on the form of underlying dynamics.

To demonstrate the insights gained from the two metrics, we extracted structural brain networks from publicly available datasets that incorporated diffusion weighted imaging (DWI) and functional magnetic resonance imaging (fMRI) modalities. Dynamical models of neural ensemble activity at macroscopic scales—the mean field model (MFM) and the LSM—can be used to simulate the node dynamics of brain networks (discussed in detail in Neurodynamical Models section). Using an optimization approach of functional connectivity distance (Naskar et al., 2021; Vattikonda, Surampudi, Banerjee, Deco, & Roy, 2016), we tune the network parameters to a regime that maximally captures the empirical resting-state functional connectivity (rs-FC). Using parameter values informed by the optimization procedure, we carry out the perturbation protocol for a select few models that span the dynamical landscape. We use the simulated time series to compute the net influence and flow for each region.

### Datasets

The generality of the two measures—*net influence* and *flow*—were validated on two separate human neuroimaging datasets: the Multimodal Imaging and Connectome Analysis–Microstructure-Informed Connectomics (MICA-MICs) dataset (available in the Canadian Open Neuroscience Platform Portal) (Royer et al., 2021) and the Cambridge Centre for Ageing and Neuroscience (Cam-CAN) dataset (Shafto et al., 2014; Taylor et al., 2017). Different parcellation schemes were used on each of the datasets to further showcase that the outcomes do not depend on the extent of brain parcellations that govern the size of the network analyzed.

#### MICA-MICs.

SC matrices derived from DWI data of 50 healthy participants (27 males; 23 females; age: 29.54 ± 5.62 years) were obtained from the publicly available MICA-MICs dataset (Royer et al., 2021). Of the multiple parcellation schemes available, data parcellated according to the Schaefer–Yeo seven-network scheme was chosen in order to study network-level distributions of net influence and flow, and to also eliminate the need for manual assignment of nodes to functional networks, which can be erroneous. Furthermore, to strike a balance between fine spatial resolution and interpretability of results, we used data parcellated into 300 regions. As elements of the SC denoted number of white matter tracts between regions, subject-level data was first log transformed to reduce the connectivity strength variance. Negative connections were then removed, and the SCs were averaged over 50 participants and thresholded using a distance-dependent consensus, a method which ensures that the group SC preserves subject-level network statistics (Betzel, Griffa, Hagmann, & Mišić, 2019).

The group-averaged resting-state functional connectivity (rs-FC) matrix was computed by Fisher *z*-transforming subject-level rs-FC matrices, carrying out an average over subjects, followed by an inverse *z*-transform back to correlation values. Only positive correlations were retained. This rs-FC was used as an empirical reference against simulated rs-FCs. We direct the reader to the original article (Royer et al., 2021) for details of data acquisition.

#### Cam-CAN.

Empirical DWI data of 40 healthy participants (19 males; 21 females; total age range: 18–38), sampled from the Cam-CAN cohort (Shafto et al., 2014; Taylor et al., 2017) chosen for an earlier work (Naskar et al., 2021) was used to construct the SC matrix. Cortical gray matter was parcellated into 150 regions of interest using the Destrieux parcellation (Destrieux, Fischl, Dale, & Halgren, 2010), and the subject-wise SC matrix was generated using an automated pipeline by Schirner, Rothmeier, Jirsa, McIntosh, and Ritter (2015), and was averaged over the 40 subjects to generate the group-averaged SC matrix. A curious reader may refer to the original article for additional details about the preprocessing steps used (Naskar et al., 2021).

The rs-FC matrix was required for determination of model parameters that best fit empirical data. We use a 150 region Destrieux-parcellated rs-FC matrix that was also used in the earlier article (Naskar et al., 2021). The rs-FC was generated by computing the pairwise Pearson coefficient between all pairs of *z*-transformed BOLD timeseries, for 40 subjects, and then averaged to generate the rs-FC used for empirical model validation.

### Neurodynamical Models

A number of neurodynamical models have been formulated to simulate whole brain resting-state activity from DTI/DWI-based structural connectomes. Some of the notable models include the vector auto regressive (VAR) model (Messé, Benali, & Marrelec, 2014; Tononi, Sporns, & Edelman, 1994), LSM (Galán, 2008; Goñi et al., 2014; Hansen et al., 2015), Wilson-Cowan oscillator (Wilson & Cowan, 1972), Kuramoto oscillator (Abeysuriya et al., 2018; Cabral, Hugues, Sporns, & Deco, 2011), and MFM (Deco et al., 2013; Hansen et al., 2015; Naskar et al., 2021; Vattikonda et al., 2016) among others. In order to depict the variety of network-dynamical interactions supported by an underlying SC, we use two models that we believe are far apart in terms of their dynamical complexities: the LSM and the MFM.

#### Mean field model.

The dynamic mean field model (MFM) is obtained by carrying out a mean field reduction (Deco et al., 2013) of the spiking neuron model (Deco & Jirsa, 2012). The MFM has also been shown to replicate features of empirical functional state transition dynamics, in addition to being able to predict time-averaged FC (Hansen et al., 2015). The dynamics of the MFM nodes are given by the following:

$${\dot{S}}_{i}=\frac{-S_{i}}{\tau_{S}}+\left(1-S_{i}\right)\gamma H_{i}+{\sigma \eta }_{i}\left(t\right)$$ (8)

$$H\left(x_{i}\right)=\frac{{ax}_{i}-b}{1-\exp\left(-d\left({ax}_{i}-b\right)\right)}$$ (9)

$$x_{i}={wJS}_{i}+GJ\sum_{j=1}^{N}C_{ij}S_{j}+I_{0}$$ (10)

where *S*_*i*_ is the NMDA synaptic gating variable of the *i*^*th*^ node, *H*(*x*_*i*_) is the firing rate function for the input *x*_*i*_, to the node *i*. Following Deco et al. (2013), the fixed parameters are the local excitatory recurrence *w* = 0.9, synaptic coupling *J* = 0.2609(nA), overall external input *I*_0_ = 0.3(nA), kinetic parameters *γ* = 0.641/1000 and *τ*_*S*_ = 100(ms), and firing rate function parameters *a* = 270(n/C), *b* = 108(Hz), and *d* = 0.154(s); *σ* = 0.001 is the noise amplitude and *η*_*i*_(*t*) is random number sampled from a normal distribution.

In contrast to the LSM, nodes following MFM dynamics can exhibit bistable and nonsymmetric steady states. We carry out a preliminary exploration of steady states by randomly initializing the system at both “low” (0 ≤ *S*(*t* = 0) ≤ 0.1) and “high” (0.3 ≤ *S*(*t* = 0) ≤ 1) initial conditions (Castro, El-Deredy, Battaglia, & Orio, 2020), and allowing a noiseless evolution of the system for 40 s, at each value of the global scaling parameter, *G*, which is a free parameter in the model. This is done for 10 trials. While the low-activity “spontaneous fluctuations” state is highly stable for lower values of *G*, increasing it pushes the system to a bistable regime. Further increase of *G* renders the spontaneous state unstable (Supporting Information Figures S2 and S3).

#### Linear stochastic model.

The LSM (Galán, 2008) is a simple neurodynamical model that can be obtained by removing the saturation function and inhibitory population of the Wilson–Cowan model (Wilson & Cowan, 1972). The dynamics follow:

$${\dot{x}}_{i}=-x_{i}+G\sum_{j=1}^{N}C_{ij}x_{j}\left(t\right)+{\sigma \eta }_{i}\left(t\right),$$ (11)

where *x*_*i*_(*t*) is the activity of region *i*, *G* is the global scaling parameter, *C*_*ij*_ is an element of the structural connectivity matrix, *σ* is the noise amplitude, and *η*_*i*_(*t*) is a random number from a Gaussian distribution with zero mean and unit variance. All nodes following LSM dynamics have two possible steady states, depending on the value of *G*: zero for 0 ≤ *G* < *G*_*stable*_ and +∞ for *G* > *G*_*stable*_. In order to keep noise from pushing the system into the divergent regime, we first determine *G*_*stable*_ by carrying out a randomly initialized noiseless simulation by varying *G* in steps of 0.01 for 20 trials. Once *G*_*stable*_ is determined, we set the noise amplitude as *σ* = *G*_*stable*_ − *G* (Hansen et al., 2015) for the parameter estimation runs of the model.

### Computational Protocol

All neurodynamical models were numerically integrated using the Euler integration algorithm (Mannella, 2002) with a time step of 1 ms. The initial conditions (*S*_*i*_(*t* = 0) for the MFM and *x*_*i*_(*t* = 0) for the LSM) for all the models were sampled from a uniform distribution in the interval [0, 1].

#### Empirical best fit parameter estimation.

The first step in the computational protocol was to identify the critical value of the scaling parameter *G* for the respective models and datasets. These simulations were carried out by numerically integrating the respective stochastic neurodynamical differential equations at a certain value of *G*, for 96 s for the MICA-MICs dataset (315.2 s for the Cam-CAN dataset). The time series were then passed through a Balloon–Windkessel hemodynamic filter (Cabral et al., 2011; Friston, Harrison, & Penny, 2003; Friston, Mechelli, Turner, & Price, 2000) for BOLD signal generation. The parameters for the model were set as per Friston et al. (2003). We then remove the initial 12 s (39.4 s for Cam-CAN) of BOLD signals to remove transients, after which the signals are downsampled to the respective *T*_*R*_ values of 0.6 s for the MICA-MICs and 1.97 s for the Cam-CAN dataset to match the sampling rate of the empirical data. The total duration of the simulation and the length of the window to remove transients were chosen to be long enough to arrive at a good representation of the resting state, and were also multiples of the respective *T*_*R*_ to make the handling of data easier. The synthesized BOLD signals were then *z*-scored and the pairwise Pearson correlation was computed to generate the simulated rs-FC. This protocol is repeated for each *G* value from 0–0.02 in steps of Δ*G* = 0.001 (MICA-MICs dataset) and from 0–0.5 in steps of Δ*G* = 0.01 (Cam-CAN dataset), with the protocol repeated for 30 trials per *G* value. The conformity of the simulated rs-FC with the empirical rs-FC was gauged through the functional connectivity distance between the two (refer to Parameter Space Identification section). This protocol helped arrive at the critical parameter value, G*, which was then used along with neighboring values of *G*, as test cases of the perturbation protocol.

#### Perturbation protocol.

The perturbation protocol for a neurodynamical model evolving on a DWI–based structural connectome comprised two major steps: an evolution to steady state, followed by an evolution to a perturbed steady state. First, the model is initialized randomly and allowed to noiselessly evolve over a period of 60 s, at the end of which the values of the dynamical variables of the models are stored as the unperturbed steady state. Second, each node is chosen as a source, *n*, and its value is reset to (1 + *α*)*x*_*n*_, and the remaining nodes of the system are initialized at their respective steady-state values. The system is then allowed to evolve for 5 s, by which it stabilizes at a new “perturbed” steady state, which was then stored. The value of *α* was fixed as −0.1 as used in Harush and Barzel (2017). The negative value of the perturbation was chosen to prevent the system from being pushed to an unstable regime through the perturbation. The differences between the unperturbed and perturbed steady-state values are used to compute the linear response matrix, using Equation 2. Simulation times were ensured to be long enough for steady-state stabilization. We carry out noiseless simulations and forego BOLD generation in the perturbation protocol, as it is sufficient for purposes of demonstration of the method. For cases where the unperturbed steady state is trivial, we manually set the steady state to a small positive value (10^−60^) prior to starting the perturbation step to avoid division by zero errors in Equation 2. The measurement of net influence through each node *i* is then given by Equation 4 and the flow through the node is measured through Equations 5, 6, and 7.

### Parameter Space Identification

Parameter space for the model for each dataset can be tuned using an approach developed by earlier studies (Naskar et al., 2021; Vattikonda et al., 2016) where the difference between the model predicted FC and the empirical FC was quantified using the FC distance measure, given by the following:

$$FCD=\frac{1}{N}\sqrt{\sum_{i=1}^{N}\sum_{j=1}^{N}{\left({FC}_{emp}\left(i,j\right)-{FC}_{sim}\left(i,j\right)\right)}^{2}}$$ (12)

The value of *G* at which minimum FCD is observed (Supporting Information Figure S1) is referred to as the G*.

### Net Influence and Flow Through Resting-State Networks

Previously computed node-level net influence and flow values from the MICA-MICs dataset were used to study how the net influence and flow values are distributed across the nodes belonging different resting-state networks (RSNs). The Schaefer300 7-Network parcellation of the MICA-MICs data allows each node from both the hemispheres to be clubbed into one of the RSNs (Vis = visual, SMot = somatomotor, DA = dorsal attention, SNVA = salience network/ventral attention, Lim = limbic, Cont = fronto-parietal control, DMN = default mode network). Once the nodes were assigned to their respective networks, they were first sorted in the descending order in terms of their net influence, after which the RSN distributions of the top 10% nodes (positive net influence values = Influencers) and bottom 10% nodes (negative net influence values = Followers) were computed. The RSN distribution of nodes with the top 10% flow values was also similarly computed.

To study whether RSNs have a balanced distribution (zero mean) of net influence and flow across its constituent nodes, we computed the sums of net influence and flow of every node in each of the seven RSNs, and averaged them by the number of nodes in the respective RSNs to arrive at the metrics of net influence and flow per node. Just like the node-level interpretation, RSNs with a positive value of net influence per node can be viewed as being influential over network dynamics, whereas those with negative values of net influence per node can be viewed as being primarily driven by the rest of the networks. Similarly, RSNs with high flow per node values can be viewed as centers that mediate information flow.

## RESULTS

*Net influence* and *Flow* were computed from simulated resting-state time series generated from the whole-brain connectome where each node can be modeled according to a neurodynamical model—mean field model (MFM) or linear stochastic model (LSM). The structural connectivity (SC) matrix for the whole-brain model was generated from the MICA-MICs and Cam-CAN databases, and resting-state functional connectivity (rs-FC) from empirical data was used to optimize model parameters (see Methods for details). In this section, we first report the results of carrying out a perturbation protocol at a node level for the MFM and subsequently estimating the net influence for each node in the whole-brain connectome. Second, we present flow distributions for the same network, which implements the functional lesion step, together with the standard perturbation protocol, and highlights brain regions that are crucial to the overall transfer of information in the brain. We investigated how this relationship changed when the free parameter *G* of MFM was varied, with G* corresponding to the critical value of *G* that best fits empirical data. The final result involves the computation of the net influence and flow per node in each of the seven resting-state networks (RSNs) in the Schaefer–Yeo parcellation to test whether the RSNs on average have nontrivial network influencing and information flow mediating capabilities. In the first two analyses, the net influence and flow are studied as a function of local neighborhood properties (node strength), whereas the final analysis involves the computation of RSN-level metrics that are then used to compare the seven RSNs. The computation of net influence and flow for other values of *G* (representative of low activity, bistable, and high activity regimes; Supporting Information Figure S2) further illuminates how the criticality of the nonlinear dynamical system is reflected in the information transfer properties. The origin of this sensitivity to *G* lies in the form of the firing rate of the MFM, which will be further discussed in the Discussion section.

While the main text presents results for the MFM evolving on the SC derived from the MICA-MICs dataset, as a demonstration of generality, the entire pipeline (except for the RSN-level analysis) was repeated for the Cam-CAN dataset running both the MFM and the LSM. These results are presented in the Supporting Information figures.

### Perturbation Unearths Asymmetries in Influence Capabilities

**Figure 2.**
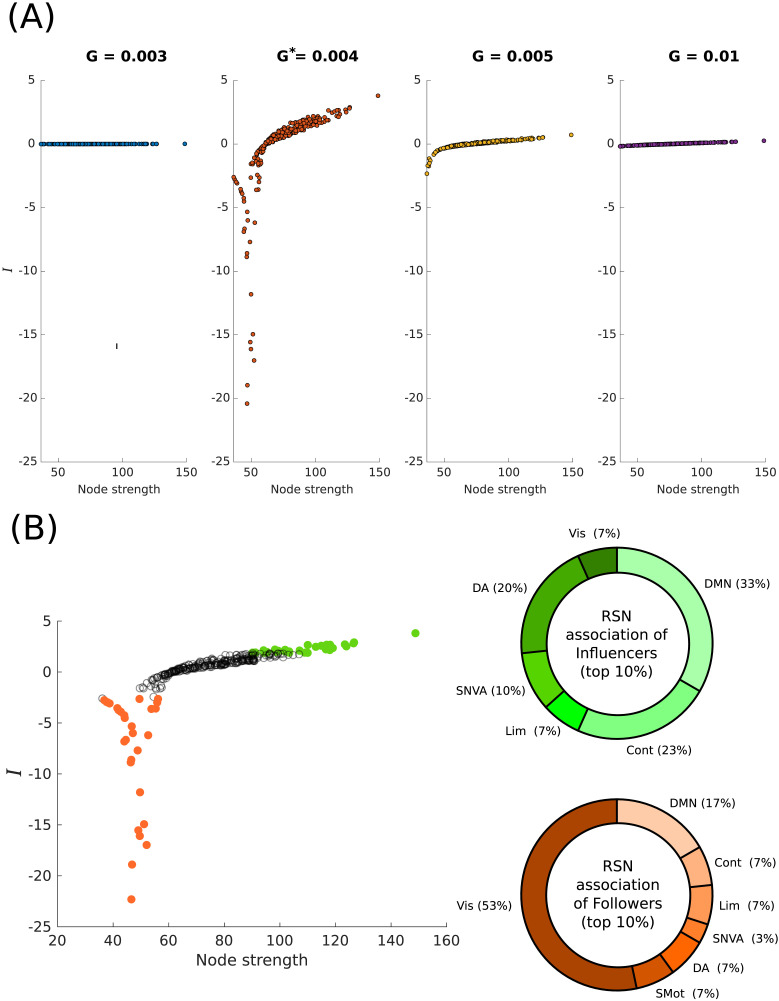
Node-level response asymmetry. (A) Response asymmetries, quantified as the net influence of nodes, *I*_*i*_ = $\sum_{m=1}^{N}$ *R*_*mi*_ − $\sum_{m=1}^{N}$ *R*_*im*_, as a function of node strengths (sum of log number of white matter tracts between regions). The values of the global scaling parameter for which the asymmetries are computed are shown in Supporting Information Figure S2. Response asymmetry is maximized at G* = 0.004 and reduces as *G* is increased. (B) Net influence distribution with node strength for G*, with nodes displaying the top 10% positive net influence values (green) and top 10% negative net influence values (orange) highlighted (*left*); the distribution of the top 10% influencer and follower nodes across the seven resting-state networks (RSNs) (*right*). Con = fronto-parietal control network; SNVA = salience/ventral attention network; DMN = default mode network; Vis = visual network; Lim = limbic network; SMot = somatomotor network; DA = dorsal attention network.

Nonzero net influence following a perturbation protocol captures the response asymmetry of a particular node of the network, and is a function of node strength. We used a group-averaged SC from the MICA-MICs dataset (Royer et al., 2021), whose elements quantify the number of white matter tracts between cortical gray matter regions. We observe that the relationship between net influence and the node strength computed from MFM simulations on the MICA-MICs SC displays a critical dependence on *G* (Figure 2). The influencer-follower hierarchy is most well defined at critical G* = 0.004. As *G* is increased, we observe that this hierarchy progressively decreases, with the response of regions to perturbations from the rest of the network becoming comparable to the response it elicits in the rest of the network through its own perturbation. The topological profile of the asymmetry, which is captured by its relation to the node strength, shows that high-strength nodes unequivocally exert a positive influence on the rest of the network. The influence of the nodes of low strength on the other hand, although negative, depends on *G*, with the most negative influence at G* shown by intermediate strength nodes. For the Cam-CAN dataset, similar dependence with *G* was observed as well (Supporting Information Figure S4).

At G*, we find that the top 10% influencers comprise nodes belonging primarily to multimodal RSNs, with a small share of unimodal (visual network) regions (7%). The top 10% followers on the other hand, show a strong unimodal representation (60%), with the highest number of top followers belonging to the visual network.

We observe a well-defined influencer-follower hierarchy with node strength in the LSM as well (Supporting Information Figure S6). We note that the net influence-node strength relationship bears a faint qualitative resemblance with that of the MFM at its respective G*, with high-strength influencers and low-strength followers. However, the form of the relationship remains robust throughout *G*, and unlike the MFM, we see no critical behavior at G* = 0.19. Instead, the hierarchy progressively increases with *G*, while maintaining its qualitative form.

### Flow is Maximized for Nodes of Intermediate Strength in the MFM

**Figure 3.**
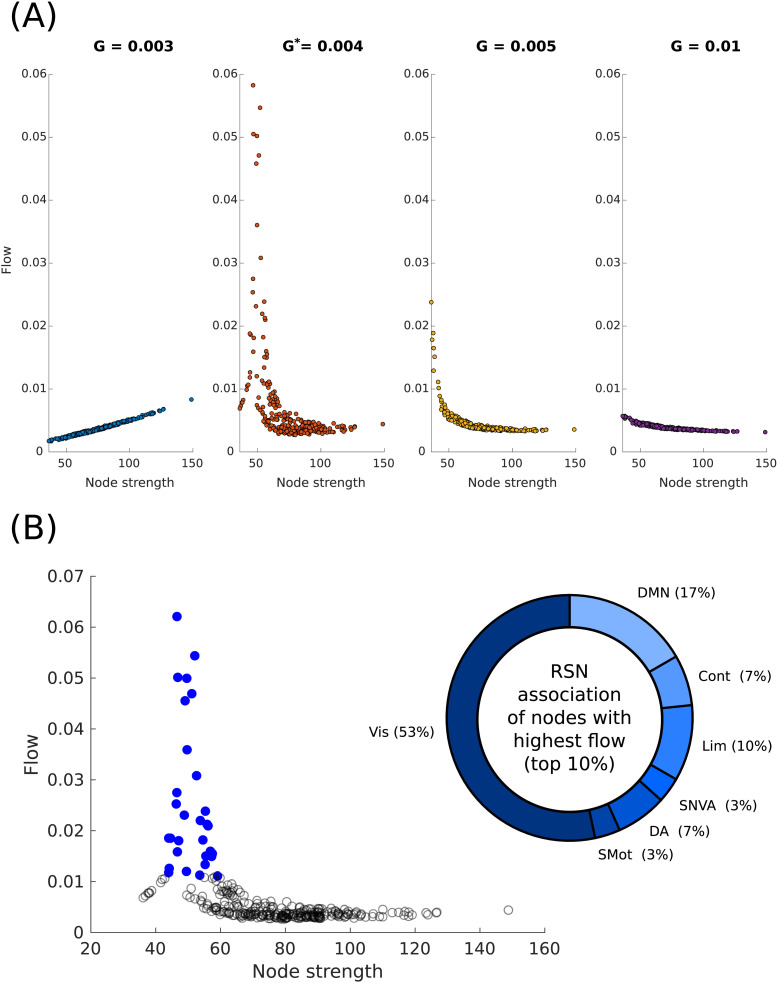
Node-level flow distribution. (A) Flow through a node, quantified as the effect of freezing the activity of node on the magnitude of responses elicited on the rest of the network, as a function of node strengths. The values of the global scaling parameter for which the flow values are computed are shown in Supporting Information Figure S2. Flow is maximized for low to intermediate strength nodes when G* = 0.004, but approaches a low-strength flow dominance as *G* is increased. (B) Flow distribution with node strength for G*, with nodes displaying the top 10% flow values highlighted (*left*); the distribution of the top 10% nodes with the highest flow across the seven RSNs (*right*).

The flow structure (flow-node strength relationship) computed for the MFM shows a clear dependence on the scaling parameter *G* (Figure 3). Flow patterns varied from a high-strength dominated flow structure for *G* < G* to an increasingly low-strength dominated flow structure for *G* > G*. At the critical G*, we find that flow is not strictly dominated by low-strength nodes, and is rather maximized for nodes that have an intermediate strength. We find that as *G* is slightly increased, the flow quickly becomes dominated by low-strength nodes.

We also observe that for *G* ≥ G* the regions displaying high flow also display a net negative perturbative influence on the network. This relation between the response asymmetry and flow structure is not observed for *G* < G*. Identical pipeline run on the Cam-CAN dataset (Supporting Information Figure S5) yields qualitatively similar patterns, as seen in Figure 3.

At G*, we note that the top 10% highest flow nodes comprise members of all seven RSNs, with unequal shares (Figure 3B). The greatest proportion of nodes belong to the visual network, and the least contribution to the high-flow nodes comes from the salience/ventral attention network. We also note that there is an almost 50% split between the unimodal RSNs (visual and somatomotor networks) when compared to the rest of the RSNs combined. We also observe that the RSN distribution of the high-flow nodes is almost identical to the RSN distribution of the top 10% followers (Figure 2B).

The results of the MFM are in contrast to that of the LSM, which shows a clear low-strength-dominated flow structure throughout *G* variation (Supporting Information Figure S6).

### Average Net Influence and Flow of RSNs

To investigate whether network-dynamical interactions endow different large-scale RSNs (defined in the Schaefer–Yeo seven network classification; Schaefer et al., 2018; Yeo et al., 2011) with unequal capabilities to influence other functional networks or mediate information flow in the brain, we checked whether the RSNs have a nontrivial net influence and flow per node (see Methods). We find that out of the seven RSNs, only the visual and limbic networks can be classified as followers. The visual network is the strongest follower, and is also the RSN that plays the strongest role in mediating information flow between arbitrary nodes, whereas the somatomotor network has the least amount of flow. The remaining five RSNs are all influencers, with the salience/ventral attention network having the strongest net influence per node.

### Model Dependence of Network-Dynamical Interactions

As mentioned in Methods, the response matrix is a construct that captures steady-state deviations upon perturbation, thereby being dependent on the form of dynamics of the system. Thus, a change in the dynamical model used should be reflected in the distributions of the net influence and flow of regions, which are derived from the response matrix. Despite both the LSM and the MFM being capable of individually conforming to empirical data (Supporting Information Figure S1), we find that the models are greatly different in terms of their dynamical and information transfer characteristics, as demonstrated by comparing their respective net influence and flow distributions (Figures 2 and 3, and Supporting Information Figures S6 and S7).

## DISCUSSION

In this article, we propose a scale-invariant approach, inspired by previous work in network science (Harush & Barzel, 2017), that quantifies the interaction between brain network structure and the dynamics exhibited by constituent nodes. We achieve this by studying the response of individual brain regions and subnetworks to controlled perturbations. We apply this technique on simulated data generated from empirically derived whole-brain connectomes where constituent nodes follow mean field model (MFM) and linear stochastic model (LSM) dynamics, for purposes of demonstrating its applicability. We find that the dynamically rich MFM is capable of exhibiting a wide range of qualitatively different network-dynamical interactions that can be captured in the form of response asymmetries and flow structures, as a function of the topological features of the network (node strength) and the model parameters (Figures 2 and 3). We could replicate similar results when a different cohort and parcellation scheme was used to derive the structural connectivity (Supporting Information Figures S4 and S5). We also observe that on using the LSM to simulate resting-state brain dynamics, the computed net influence and flow greatly differ from that of the MFM (Supporting Information Figures S6 and S7). This illuminates the implications of node dynamics on information flow, despite their abilities to synthesize data that conform to empirical observations.

Beyond the demonstration on a specific model, the broad objective of this study is to showcase the applicability of this technique on systems of any scale, as long as it is stable and responsive to external perturbations. We focus on the usefulness of the response matrix, which can be easily populated using experimental or clinical stimulation data without a priori knowledge about the dynamics. This matrix can then be used to provide neurodynamical insights through derived metrics that can have considerable use in experiment and intervention design. Moreover, the inverse problem of what dynamical form can lead to observed response asymmetries and flow patterns can greatly narrow down the search for candidate models of neural dynamics. In contrast to measures such as Granger–Geweke causality (Dhamala, Liang, Bressler, & Ding, 2018), which use statistical covariations as a proxy for information flow, our proposed approach directly quantifies information as changes in neural activity, which is a direct product of the network architecture and dynamical complexity of the system.

### Response Asymmetry Along the High-Low Strength Axis

The significance of the core-periphery axis has been pointed out by studies on various fronts (Bassett et al., 2013; Battiston, Guillon, Chavez, Latora, & de Vico Fallani, 2018; Castro et al., 2020; Gollo et al., 2017; Gollo, Zalesky, Hutchison, van den Heuvel, & Breakspear, 2015; Harriger, van den Heuvel, & Sporns, 2012; Mišić et al., 2015; van den Heuvel, Kahn, Goñi, & Sporns, 2012). Importantly, studies have also shown that neuropathological conditions are widely associated with changes to the structural core (Crossley et al., 2014; Fornito, Zalesky, & Breakspear, 2015; Stam, 2014; van den Heuvel et al., 2013), as opposed to the periphery. The net influence distributions reported in this work show a clear response asymmetry of nodes along the high-low strength axis, with low-strength *follower* nodes predominantly exerting a negative influence on the network and high-strength *influencer* nodes exerting a positive influence on the network. This is observed in both the MFM and the LSM (Figure 2; Supporting Information Figures S4 and S6). A positive net influence translates to the ability of a node, upon perturbation, to elicit a greater response on the rest of the network, compared to the network’s effect on it. Thus, any perturbation in the activity of a high-strength region (that can possibly result from a neuropathology, exogenous stimulation, etc.) will have considerable effects on the rest of the network. Our results thus agree with previous studies and suggest that the regions in the core (which by definition will comprise a subnetwork of high-strength nodes) make ideal high impact targets for disruption of network function. Asymmetries in undirected (symmetric) networks have previously been studied in the context of sender-receiver hierarchies arising from effective connectivity patterns by Seguin et al. (2019). We investigate how response asymmetries as those reported in this work can arise from an otherwise undirected network in the Supporting Information Figure S8. Figure 2B additionally shows that the top influencers and followers are primarily associated with multimodal and unimodal RSNs, respectively. Such a functional separation can arise from possibly nonoverlapping strength distributions of the nodes of different RSNs, since the net influence has a well-defined relationship with the node strength.

Neurodynamical models have been shown to display critical regimes associated with the onset of dynamical richness and spatiotemporal organization of nodes into RSNs (Deco & Jirsa, 2012; Deco, Jirsa, & McIntosh, 2011; Haimovici, Tagliazucchi, Balenzuela, & Chialvo, 2013). Models that operate at these critical working points are also found to conform to a great degree to empirical resting-state data. Our results show that the criticality of such models are also reflected as stark qualitative changes in information transmission and dynamical influence characteristics, captured through the metrics of net influence and flow (Figures 2 and 3).

### Flow Hubs Are Mediators of Information Transfer

Modeling communication or signaling dynamics in brain networks is an important problem in neuroscience, as communication underlies the healthy function of a functionally heterogeneous entity like the brain (Avena-Koenigsberger et al., 2018; Graham, Avena-Koenigsberger, & Mišić, 2020). Flow is a dynamics-dependent quantity that adds a dimension of complexity to static network–based measures of quantifying roles in communication such as communicability centrality (Estrada, Higham, & Hatano, 2009). The utility of flow lies in its ability to identify nodes through which the maximum amount of information flows, making them ideal targets to study or intervene and modulate the spread of network perturbations.

The calculation of flow using Equation 7 condenses the overall effect of the functional lesion of ‘i’ over all possible perturbations in the network, and thus equivalently all possible information transfer scenarios. The lesion of high-flow nodes thus have the greatest impact on information flow in an active network for the given dynamics. This can have particular applications in designing interventions to limit the impact of perturbations to healthy brain function that can be brought about by neuropathologies, which can eventually result in a “new normal” (the disease state), similar to the perturbed steady state in this study. Flow can also find its use in the study of regions facilitating seizure propagation in epilepsy, using whole-brain epileptor models (El Houssaini, Bernard, & Jirsa, 2020; Jirsa et al., 2017; Proix, Bartolomei, Guye, & Jirsa, 2017).

Our results show that, for the MFM, flow is maximum for nodes of intermediate strength (Figure 3). Comparing these nodes to the strength distribution (Supporting Information Figure S2B) shows us that these regions are also in much greater abundance than the extreme low/high-strength nodes. Based on Gollo et al. (2015), the abundance of these intermediate strength high-flow nodes suggest that they fall into the “feeder” category, which possibly bridges the low-strength to high-strength nodes and displays fast dynamics. It is also well established that the core and peripheral nodes have distinct information processing functions (Bassett et al., 2013; Gollo et al., 2015) and constantly communicate with each other. In line with this, the intermediate strength dominance of flow in the empirical best fit model also suggests a polysynaptic parallel high-low-strength communication pathway. This can be alternatively viewed as a bidirectional *high-intermediate-low* pathway, with the intermediate strength nodes playing a central role. The low-strength node dominated flow structure observed in the LSM is in stark contrast to the intermediate strength node dominance of the MFM. Such a flow distribution suggests that the LSM supports a *high-low-high* information flow pattern, which would suggest that the low-strength nodes are simply relay points in the communication process, instead of being a center of information processing like the high-strength nodes. The MFM thus seems to be a better model in maintaining empirical conformity along with realistically plausible information flow patterns.

It must be noted that, in contrast to the three categories of flow patterns reported in Harush and Barzel (2017), with the MFM, we observe a highly nonlinear flow distribution, which further depends critically on *G*. The origin of this observation lies in the nonlinear firing rate of the MFM (Equation 9), which prevents the MFM from being written down in the general form of equations used in Harush and Barzel (2017), which is important for not only there being three primary forms of flow structure (degree-averting, degree-driven, homogeneous), but also for the lack of dependence on any scaling of the underlying connectivity matrix. In the case of the LSM, which can easily be recast into the general form, we observe that the qualitative form of the flow distribution is degree-averting and independent of *G*. By replacing the nonlinear firing rate of the MFM with a linear firing rate function determined using known bounds of the dynamical variable *S* to arrive at bounds of the function, the MFM equations can be made to conform to the general form used in Harush and Barzel (2017), in which case the *linear* MFM will also display one of the three primary flow distributions as well as *G* independence. This is shown in Supporting Information Figure S9, where the MFM is shown to have a degree-driven flow distribution, similar to the case where *G* < G* in Figure 3A.

The observation that regions with high flow are also likely to show a net negative response asymmetry, that is, response to external stimulations is greater than response elicited, is a fallout of the flow’s dependence on the linear response matrix. The activity of regions with a high negative influence can be altered more by perturbations originating from the rest of the network when compared to that of regions with lesser negative influence. This directly translates to a greater contribution by these negative influence regions to the response of all nodes to arbitrary perturbations.

### RSN-Level Analysis Offers Network-Dynamical Insights Into Function

**Figure 4.**
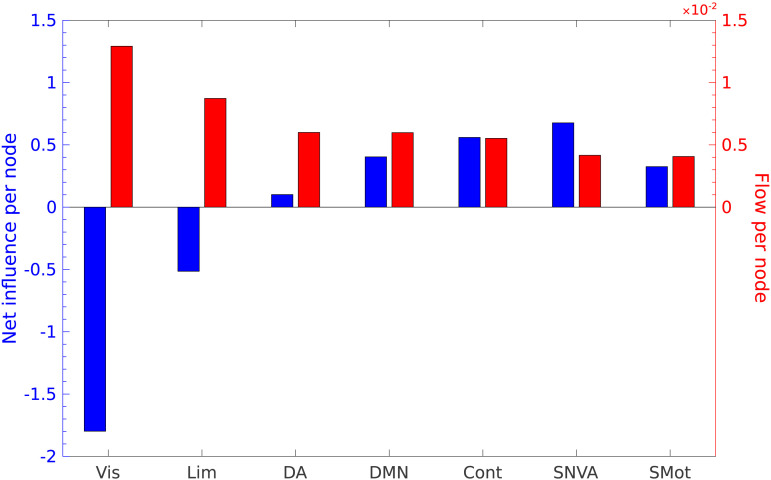
RSN-level response asymmetry and flow. The net influence per node (blue) and flow per node (red) in each of the seven RSNs in the Schaefer–Yeo parcellation. On average, nodes in the visual network have the highest flow and are also strong followers, whereas the nodes in the salience/ventral attention network comprise strong influencers.

The RSN-level analysis of net influence and flow (Figure 4) shows that the visual network is a strong follower network, with its nodes having the highest capability of mediating information flow. This result would imply that while regions of the visual network are strongly driven by the other networks (primarily multimodal) the high value of flow would imply that visual information greatly bolsters the information flow between two arbitrary nodes. This can be visualized as follows: the final response at a target node is the sum of response contributions from all the nodes in the path leading from the perturbation source, and the visual network has the highest contribution to the response compared to all other RSNs. This result could point toward a network dynamical perspective to the role of visual information in learning (Lindner, Blosser, & Cunigan, 2009), memory (Grundgeiger et al., 2013), and even its ability to influence multisensory processing, as in the McGurk (McGurk & MacDonald, 1976) and ventriloquist effects (Bruns, Liebnau, & Röder, 2011; Radeau & Bertelson, 1974). We also observe that the greatest dynamical influence on the network in this case is exerted by the salience/ventral attention network (SNVA). This result offers a possible network-dynamical take on Menon’s work on the triple network model, which discusses the role of the SNVA in influencing the engagement and disengagement of the default mode network (DMN) and central executive network, the failure of which results in major neuropathologies (Menon, 2011). It is also interesting to note that the DMN, which is located at one extreme of the principal functional gradient (Margulies et al., 2016), neither exerts the greatest influence on the network, nor does it mediate information flow to the greatest degree. This suggests that the “network-dynamical interaction gradient” offers a novel perspective of brain organization in terms of dynamical influence and interregional communication centralities, not previously captured by functional gradients.

To summarize, the net influence and flow are attributes of nodes in a network, arising from network-dynamical interactions, that effectively capture their (1) perturbative impact and (2) role in communication, respectively. While perturbation of the influencer (high net influence) nodes exerts the maximum impact on the network, the high-flow nodes are responsible for the transfer of these perturbations to different network targets and contribute the most to the communication process. It is thus possible to limit the extent of this network-wide impact from attacks to the influencer nodes by designing interventions that target high-flow nodes instead, which help contain undesirable and possibly harmful perturbations.

### Methodological Considerations

Some points have to be considered regarding this formalism, in order to truly understand its scope, and work on possible limitations. First, while the formalism is general enough to be applied on any scale, the response matrix and derived metrics are dynamics dependent. Second, the analyses here are carried out on noiseless models, as we are interested in the overall change of amplitude (activation) of signals, sufficient for a proof of concept. The formalism can be readily applied to a stable stochastic system as well. Third, the perturbations are assumed to be small enough to elicit linear responses. Fourth, although net influence and flow were computed in a dynamical regime in the neighborhood of a stable fixed point, the results are generalizable to any attractor state in node dynamics, as long as they are stable. Fifth, this method cannot be used in a setting where perturbations lead to a qualitative change in the dynamical behavior of the system (such as a Hopf bifurcation). Lastly, the present article does not explicitly showcase the scale-invariance of the method as it deals only with in silico whole-brain models. However, future extension to spike trains, local field potentials and electrocorticogram recordings are certainly possible owing to the conceptual simplicity of this technique. Additionally, we have not arrived at an analytical method for identifying the regions of high flow for nonlinear systems like the MFM in the present study. Although a large number of nonlinear models can be arranged in the general form used in Harush and Barzel (2017) for which the flow distributions can be analytically computed, many complex models extensively used in neuroscience like the MFM cannot, making a formal mathematical study into its interaction with the topology an exciting topic for future research.

This work presents a simple scale-invariant perturbative formalism that can be used to both probe the distributions of influential nodes in the network, as well as identify nodes that are central to communication processes, both of which have immense experimental and clinical importance in neuroscience. The robust theoretical framework surrounding the formalism additionally opens up possibilities of mathematically studying the communication patterns permitted by established neurodynamical models. Most importantly, the formalism is invariant to the form of the underlying dynamics and instead gathers insights purely based on the response of a perturbed system of neuronal entities, making it ideal for implementation with minimal assumptions about how the system functions.

## ACKNOWLEDGMENTS

We acknowledge the generous support of NBRC Core funds and the Computing facility. For simulations, resources from the Neuroscience Gateway (Sivagnanam et al., 2013) were used. Data collection and sharing for this project was provided by the Cambridge Centre for Ageing and Neuroscience (Cam-CAN). Cam-CAN was supported by the UK Biotechnology and Biological Sciences Research Council (Grant BB/H008217/1), together with support from the UK Medical Research Council and University of Cambridge, UK. In accordance with the data usage agreement for Cam-CAN dataset, the article has been submitted as open access. The preprocessed Multimodal Imaging and Connectome Analysis - Microsturcture-Informed Connectomics (MICA-MICs) dataset was obtained from the publicly accessible Canadian Open Neuroscience Platform Portal (Royer et al., 2021).

## SUPPORTING INFORMATION

Supporting information for this article is available at https://doi.org/10.1162/netn_a_00260. Codes used in this article can be downloaded from https://bitbucket.org/cbdl/netinfluence-flow/src/master/ (Madan Mohan & Banerjee, 2021).

## AUTHOR CONTRIBUTIONS

Varun Madan Mohan: Conceptualization; Formal analysis; Investigation; Methodology; Project administration; Software; Validation; Visualization; Writing – original draft. Arpan Banerjee: Conceptualization; Funding acquisition; Project administration; Resources; Supervision; Writing – review & editing.

## FUNDING INFORMATION

Arpan Banerjee, Ministry of Youth Affairs and Sports, Government of India, Award ID: F.NO.K-15015/42/2018/SP-V. Arpan Banerjee, NBRC Flagship program, Department of Biotechnology, Government of India, Award ID: BT/MED-III/NBRC/Flagship/Flagship2019.

## Supplementary Material



## TECHNICAL TERMS

Perturbation: Controlled change in the neural activity of a region, which results in changes in activities of other network nodes.
Net influence: A metric capturing the difference between a region’s influence on the network and the network’s influence on the region.
Flow: A metric that captures the role of a region in mediating the flow of activities between all possible sources and targets.
Response asymmetry (between two regions): Inequality in the response of a region upon perturbation of another, compared to when the source and target are reversed.
Source: The node whose activity is changed, i.e., the origin of perturbations.
Response: A resultant change in the neural activity of a region, brought about by perturbation of one or more network constituents.
Target: The node of interest which *responds* (with a change in neural activity) to a perturbation of the source.
Linear response matrix: A matrix populated with the magnitude of change (difference) in a region’s activity before and after perturbation.

## Supporting Information

https://bitbucket.org/cbdl/netinfluence-flow/src/master/
