# Supplementary material for: A perturbative approach to study information communication in brain networks
Source: Netw Neurosci. 2022 Oct 1;6(4):1275–95. doi: 10.1162/netn_a_00260 (PMC11117119; doi:10.1162/netn_a_00260)
Supplement: Supplementary file 1 [file netn-6-4-1275-s001.pdf]

Fig. S1

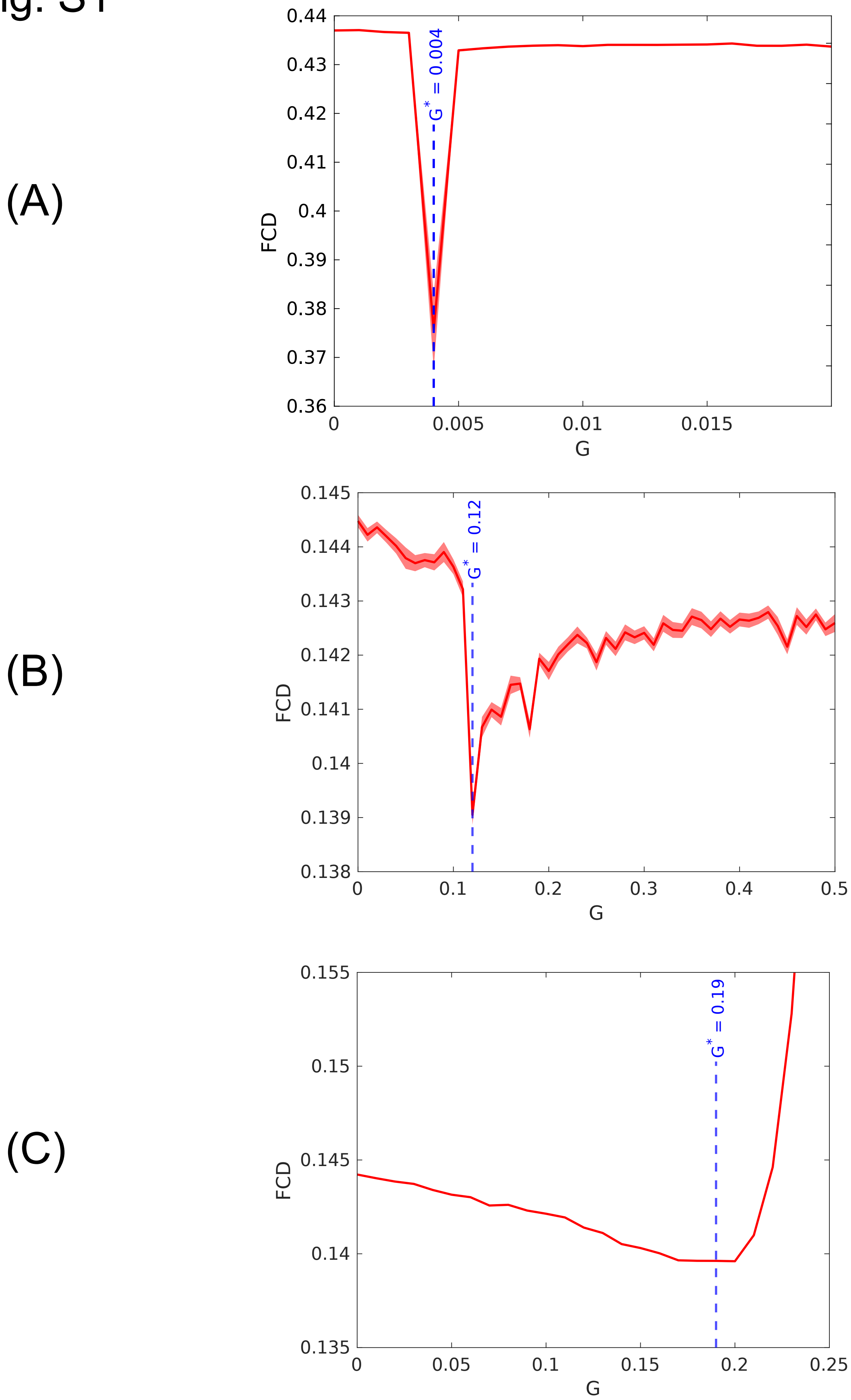

Figure S1. Identification of model parameters that maximize similarity to empirical observations
Identification of the model which best matched with the empirical ground-truth is depicted as a plot of the Functional Connectivity Distance (FCD) as a function of the free model paramter G that scales the structural connectivity (SC). The parameter value which corresponds to the best fit model minimises the FCD between the model FC and the empirical FC, and is denoted by the dashed line. The figures display the critical parameter values for **(A)** Mean Field Model (MFM) evolving on the MICA-MICs Schaefer 300 SC; **(B)** MFM evolving on the Cam-CAN SC; and **(C)** Linear Stochastic Model (LSM) on the Cam-CAN SC. Solid red line denotes the mean over trials and the red shade denotes the standard error of mean over 30 trials [Musall, 2020].

Fig. S2

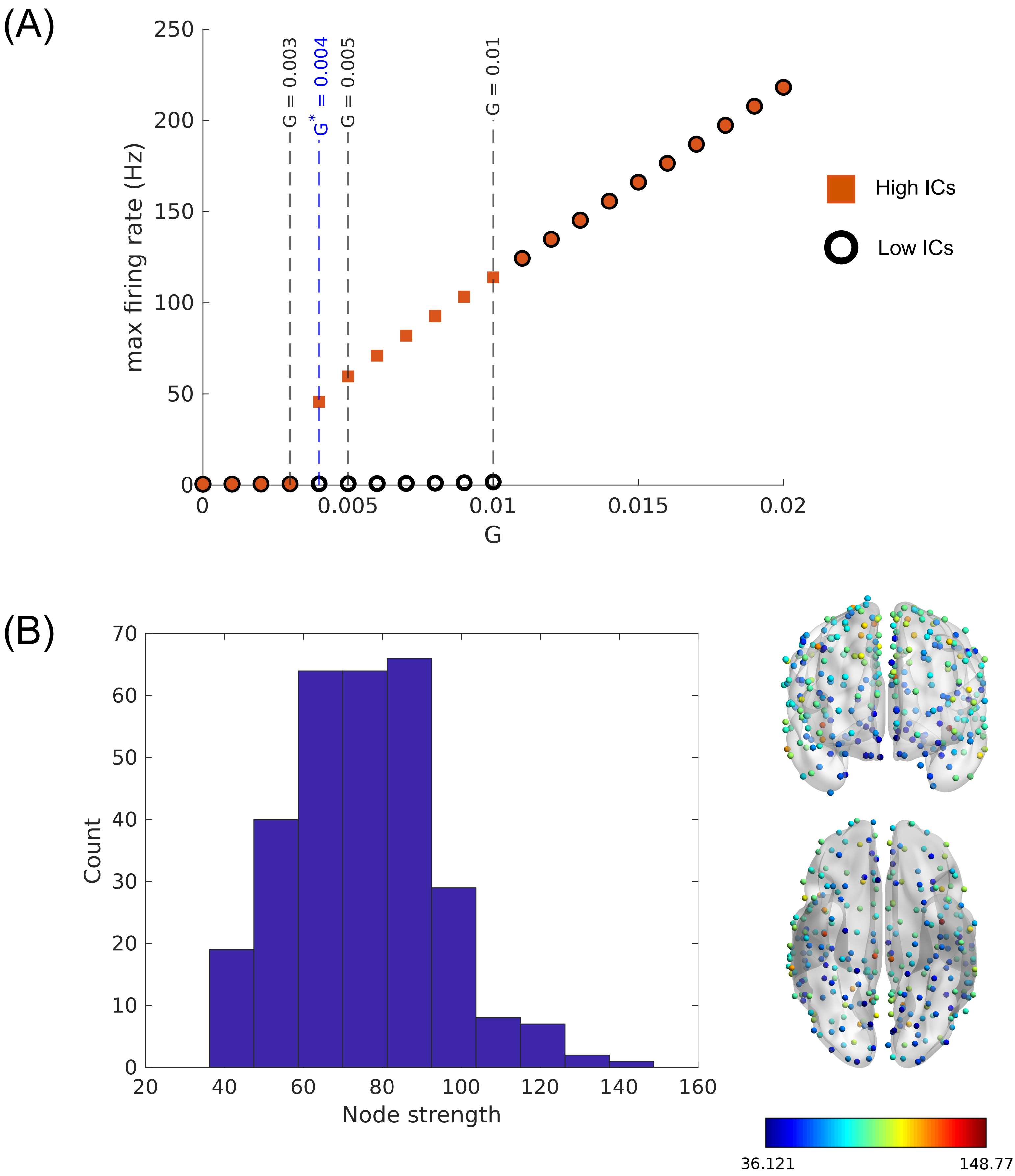

Figure S2. Mean Field Model (MFM) dynamical landscape for the MICA-MICs dataset
(A) The figure shows the dynamical landscape of the MFM as a function of the global scaling parameter *G* (orange squares = High initial conditions(ICs) ($0.3 \leq S(t{=}0) \leq 1.0$), black rings = low ICs($0 \leq S(t{=}0) \leq 0.1$)), quantified as the maximum firing rate among all the nodes in the network. Figure shows results for 10 random trials at each value of *G* ($\Delta G = 0.001$). The values of *G* for which the net influence and flows are calculated are marked by the dashed lines. The blue dashed line corresponding to $G^* = 0.004$ is the value of *G* for which the model maximally conforms to empirical data. **(B)** Node strength (sum of the log of number of white matter tracts between the node and its neighbours) distribution of the MICA-MICs dataset (*left*) with 300 nodes of the Schaefer-300 atlas, and associated BrainNet plots where colour maps to node strength [Xia et al., 2013] (*right*).

Fig. S3

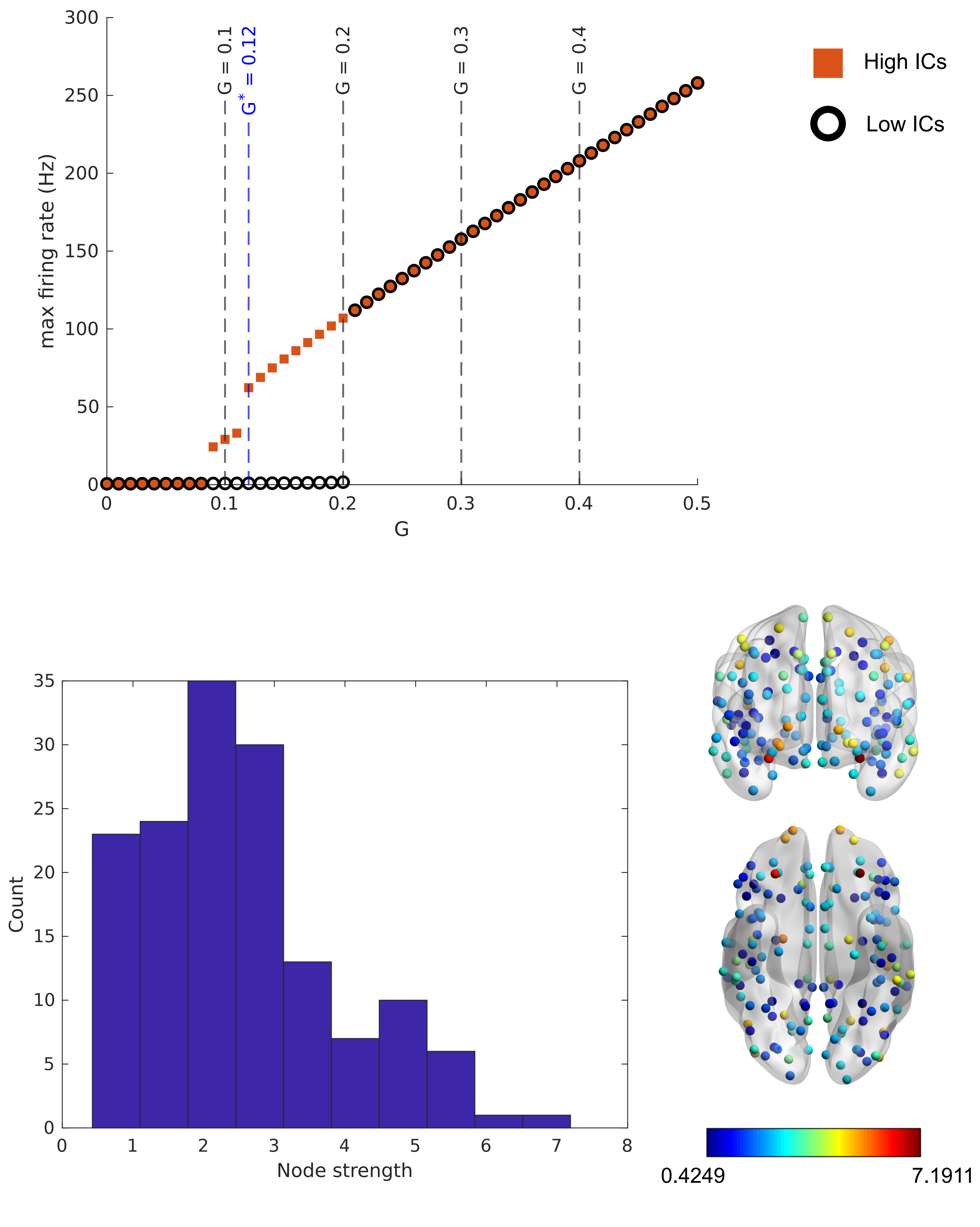

Figure S3. MFM dynamical landscape for the Cam-CAN dataset

(A) The figure shows the dynamical landscape of the MFM as a function of the global scaling parameter *G* (orange squares = High initial conditions(ICs) (0.3 ≤ S(t=0) ≤ 1.0), black rings = low ICs(0 ≤ S(t=0) ≤ 0.1)), quantified as the maximum firing rate among all the nodes in the network. Figure shows results for 10 random trials at each value of *G* (ΔG = 0.01). The values of *G* for which the net influence and flows are calculated are marked by the dashed lines. The blue dashed line corresponding to G^* = 0.12 is the value of *G* for which the model maximally conforms to empirical data. **(B)** Node strength (sum of the number of white matter tracts between the node and its neighbours) distribution of the Cam-CAN dataset (*left*) with 150 nodes of the Destrieux atlas, and associated BrainNet plots where colour maps to node strength (*right*).

Fig.S4

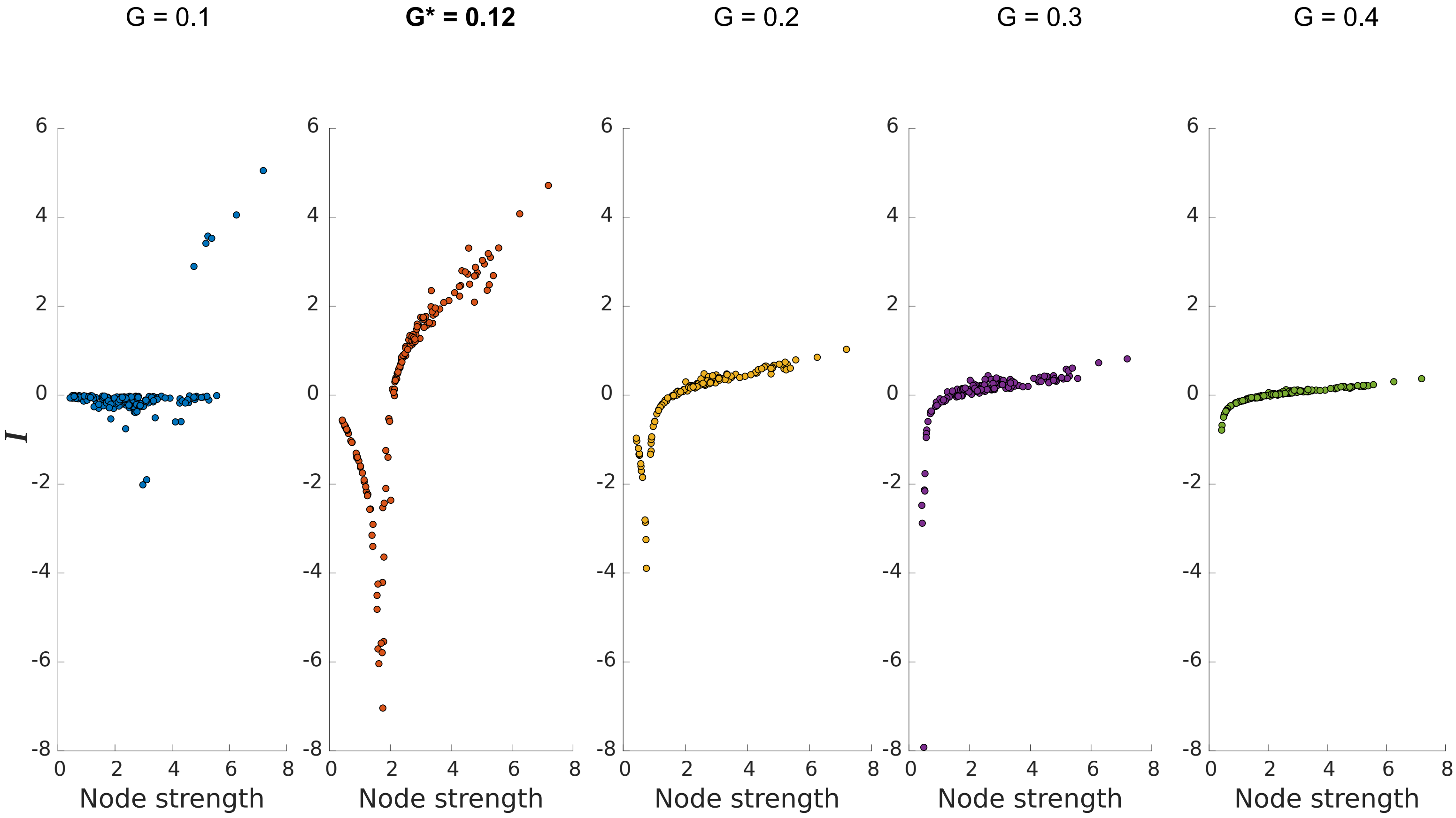

Figure S4. Response asymmetry in the Cam-CAN dataset

Response asymmetries, quantified as the net influence of nodes, I, as a function of node strengths, for nodes belonging to the Cam-CAN dataset. The values of the global scaling parameter for which the asymmetries are computed are shown in Fig.S3. Response asymmetry is maximized at $G^* = 0.12$ and reduces as G is increased.

Fig.S5

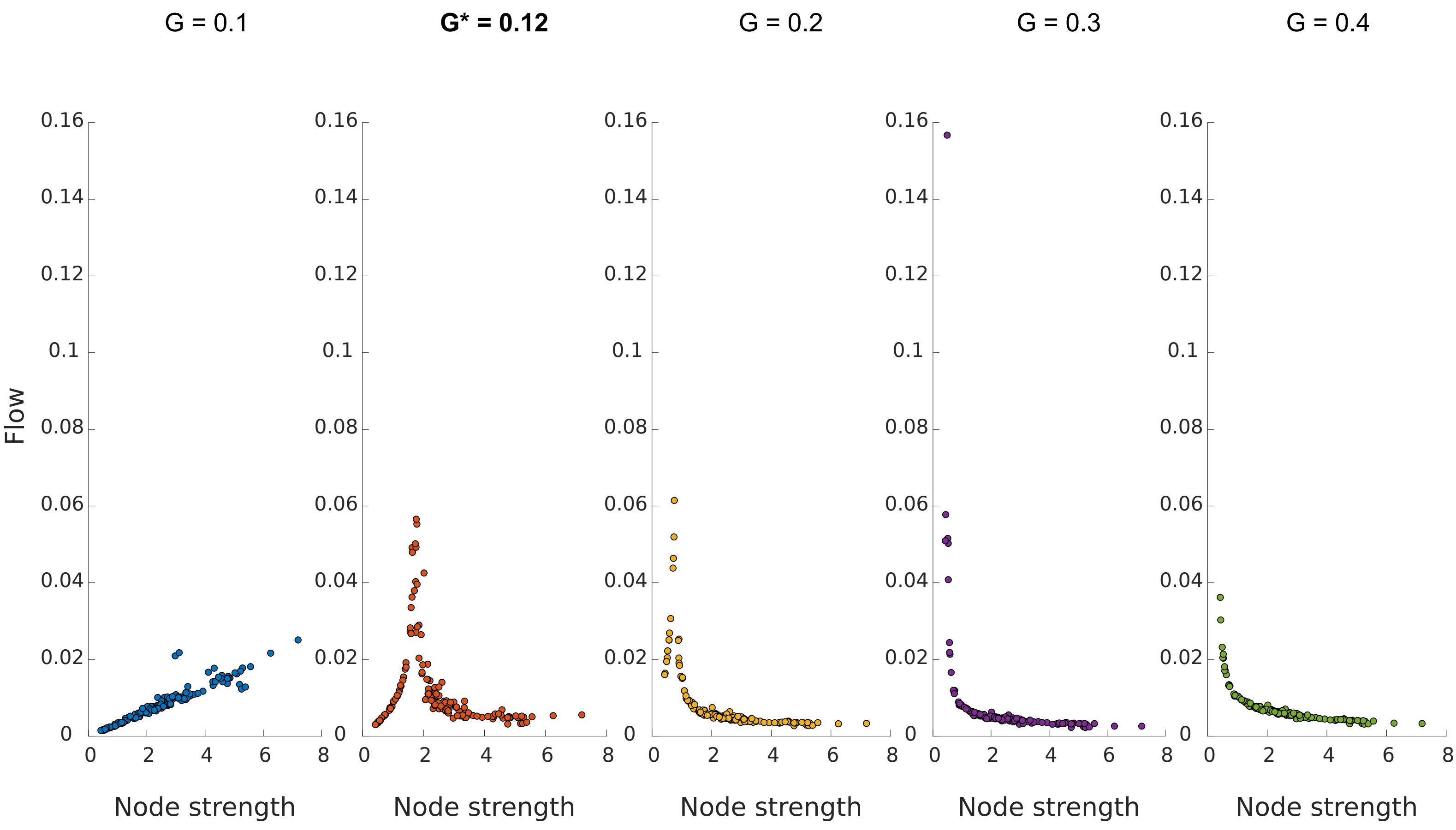

Figure S5. Node-level flow structure in the Cam-CAN dataset

Flow through a node, quantified as the effect of freezing the activity of node on the magnitude of responses elicited on the rest of the network, as a function of node strengths, for nodes belonging to the Cam-CAN dataset. The values of the global scaling parameter for which the flow values are computed are shown in Fig.S3. Flow is maximized at intermediate strength nodes for $G^* = 0.12$, but moves further towards the low strength nodes as G is increased.

Fig. S6

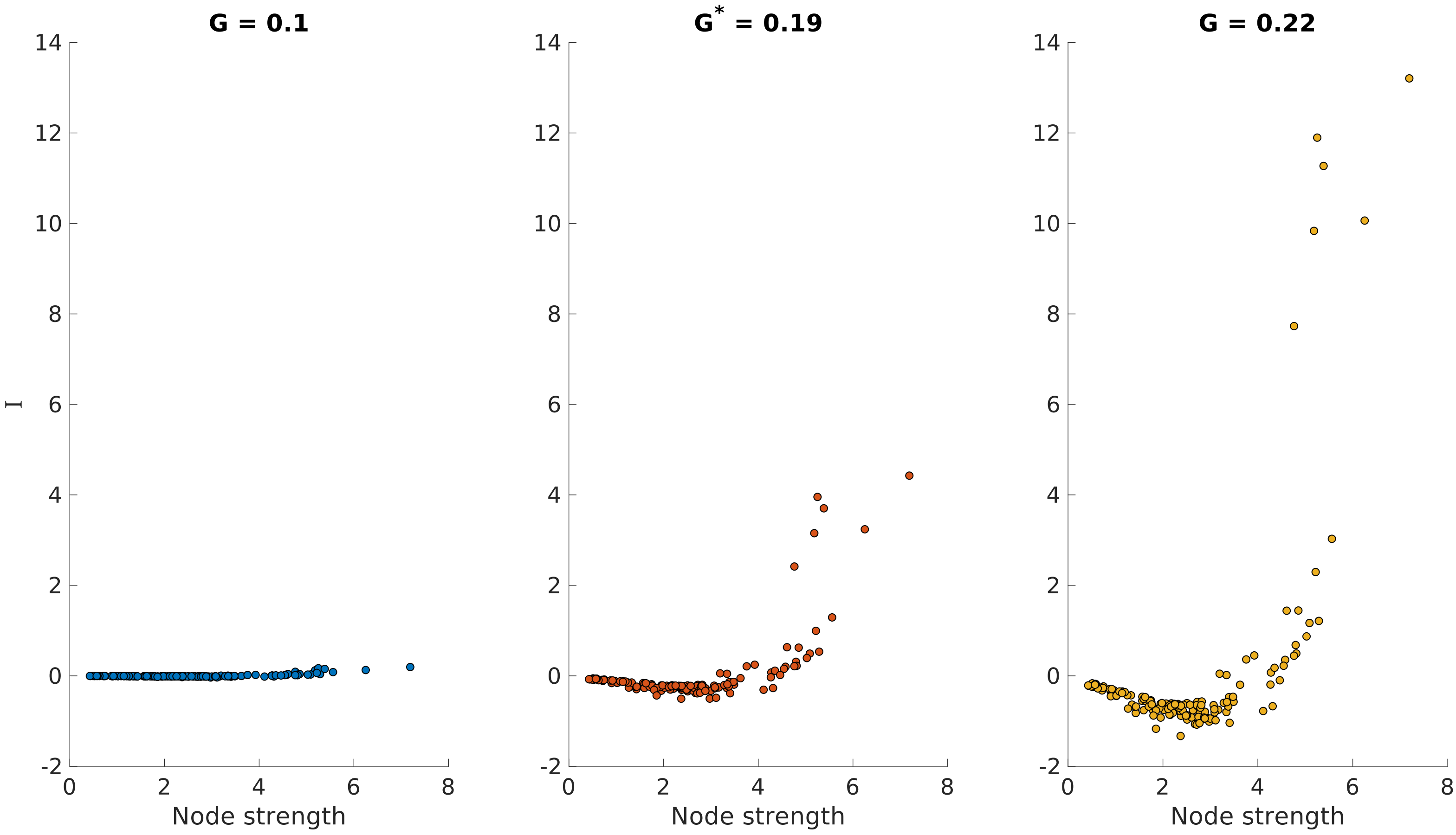

Figure S6. Response asymmetry for the LSM on the Cam-CAN dataset

Response asymmetries, quantified as the net influence of nodes, I, as a function of node strengths, for nodes following Linear Stochastic Model (LSM) dynamics, evolving over the SC derived from the Cam-CAN dataset. The values of the global scaling parameter for which the flow values are computed are centred around the empirical best fit for the LSM, $G^* = 0.19$, as shown in Fig.S1C. Similar to the nodes following Mean Field Model (MFM) dynamics at its respective G^*, we note that the LSM nodes also display an influencer-follower hierarchy along the core-periphery axis. However, unlike the MFM system, the response asymmetries progressively increase with G, and is not maximized at G^*.

Fig. S7

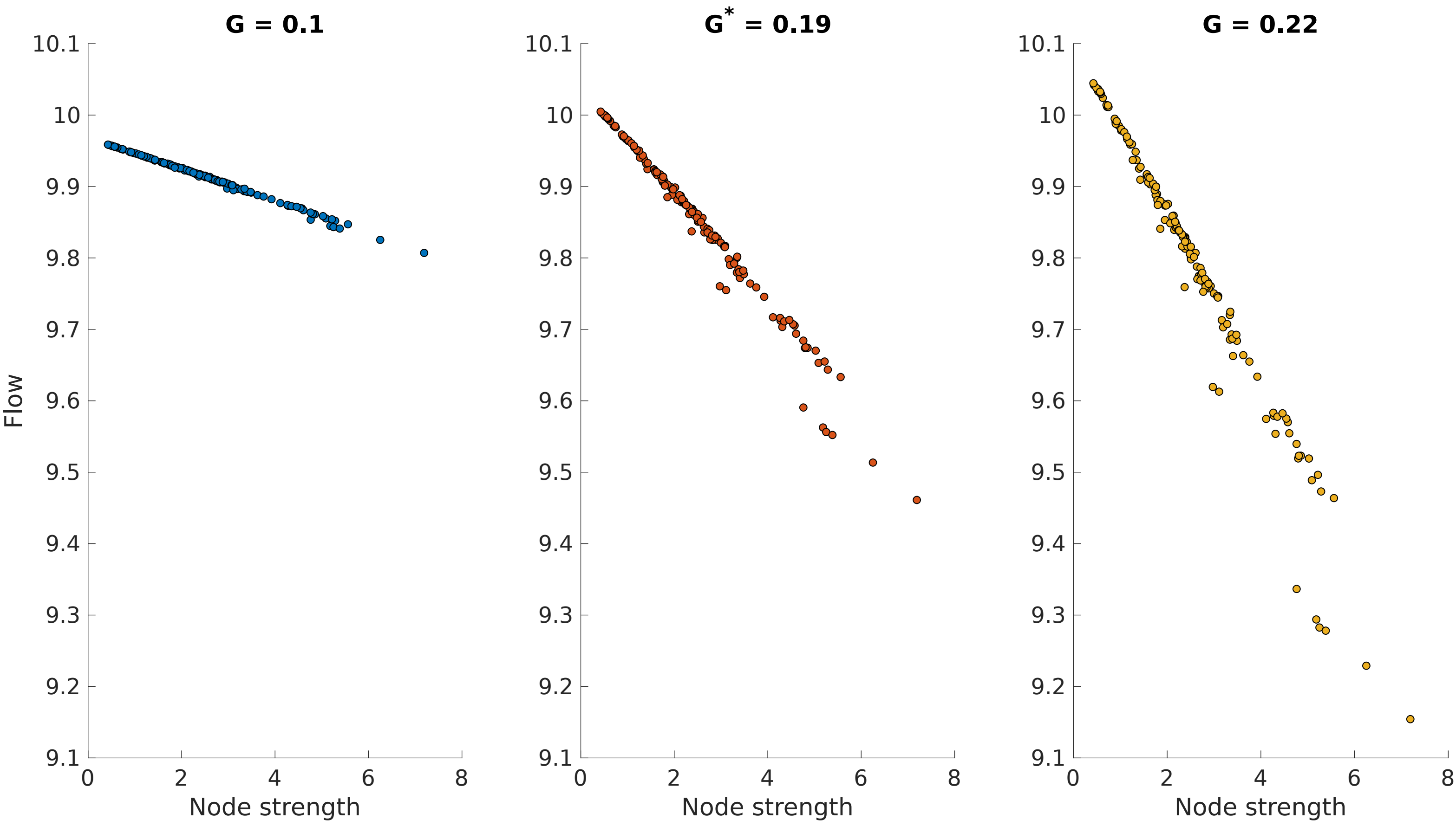

Figure S7. Node-level flow distribution for the LSM on the Cam-CAN dataset
Flow through a node as a function of node strengths, for nodes following LSM dynamics, evolving over the SC derived from the Cam-CAN dataset. The values of the global scaling parameter for which the flow values are computed are centred around the empirical best fit for the LSM, G^{*}= 0.19 , as shown in Fig.S1C. The flow is clearly periphery dominated for all the values of G, and the range of the distribution of flow values increases with G.

Fig. S8

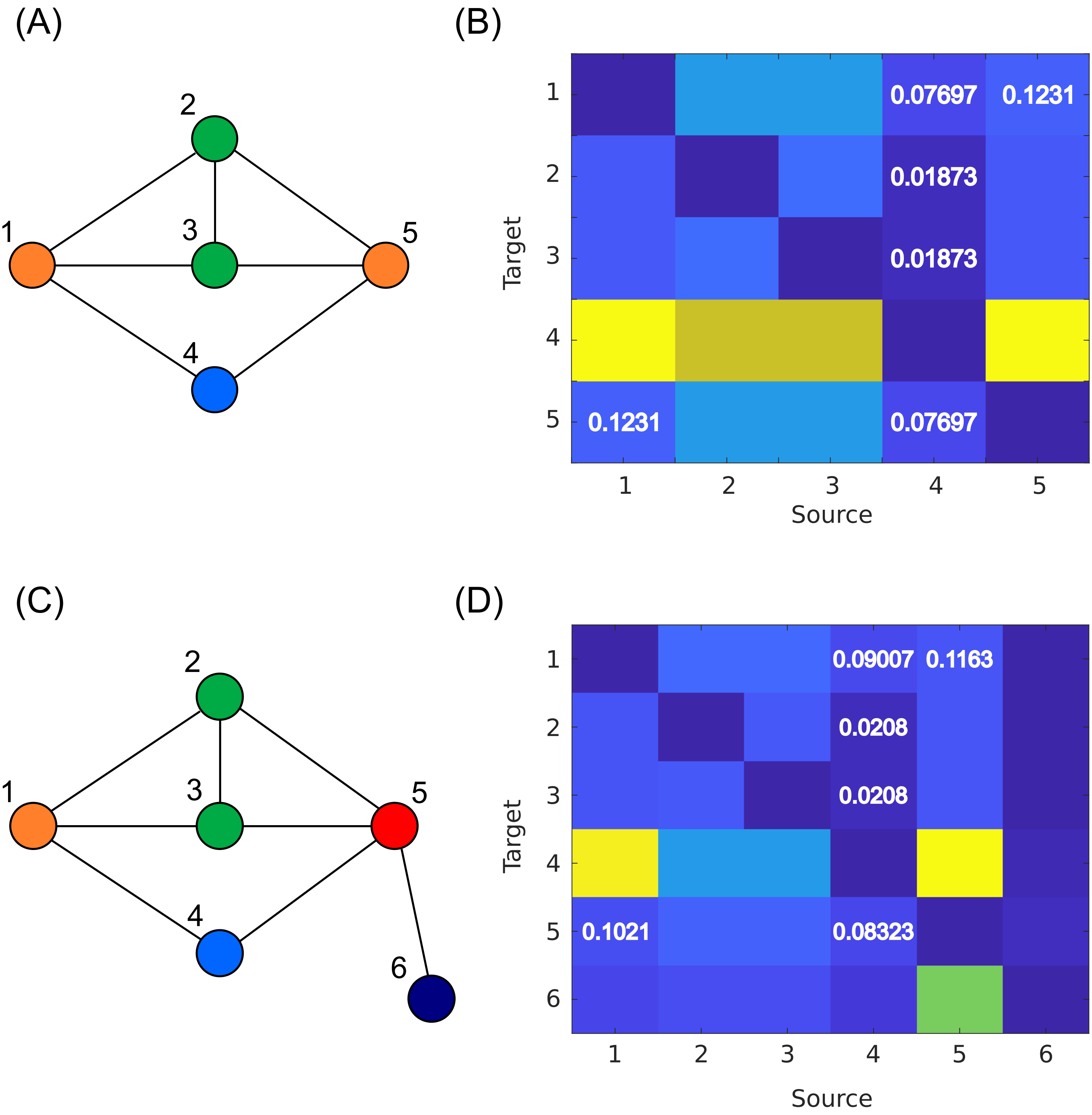
(A)
1
2
3
4
5
(B)
0.07697
0.1231
0.01873
0.01873
0.1231
0.07697
Target
Source
1
2
3
4
5
(C)
1
2
3
4
5
6
(D)
0.09007
0.1163
0.0208
0.0208
0.1021
0.08323
Target
Source
1
2
3
4
5
6

Response asymmetry: a direct consequence of network dynamical interactions

Although it seems intuitive to expect the response of directly connected bi-directional weight symmetric nodes upon perturbation to be equal, this study provides clear evidence to the contrary. In order to clearly illustrate the origin of this asymmetry, we design a simple toy network as depicted in Fig.S8. on which we run a similar perturbation protocol using the MFM (with $G = 1$), as used in the empirically derived brain networks.

In Fig.S8A, the response elicited by region ***1*** on ***2***, although directly connected, is not solely a direct influence, but a product of the perturbation reaching ***2*** through all possible paths from ***1*** to ***2***, i.e. it is a network effect. The perturbation from ***1***, in addition to reaching ***2*** through the direct edge connecting them, also traverses through the rest of the network, via ***1***'s other outgoing edges. The perturbation, upon reaching a node, causes a change in its activity, and this change is then transmitted to successive neighbourhoods. Thus, the perturbation from ***1*** impinges upon ***2*** through all of ***2***'s incoming edges. This implies that the response of a region depends on its local neighbourhood, and not on a single path between source and target. Nodes with similar neighbourhoods should thus respond similarly to a perturbation. We see this clearly in Fig.S8B, where nodes ***1*** and ***5***, and nodes ***2*** and ***3*** have similar local neighbourhoods, as a result of which the perturbation of node ***4*** elicits a similar response on the pairs.

An addition of a sixth node which is connected to node ***5*** (Fig.S8C) breaks the similarity of local neighbourhoods of ***1*** and ***5***, and we thus see that an asymmetry arises (response of ***5*** upon ***1***'s perturbation is unequal to the response of ***1*** to ***5***'s perturbation: Fig.S8D). The dynamical relation of this asymmetry borne out of local neighbourhood variability can be attributed to the form of the interaction terms in the dynamics, which for most neurodynamical models depend only on the immediate neighbourhoods of nodes. A dynamical form in which a region's interaction with the network extends to beyond its immediate neighbourhood would imply that response asymmetry would arise with variability of not just the local neighbourhood, but the extended neighbourhood instead.

Fig. S9

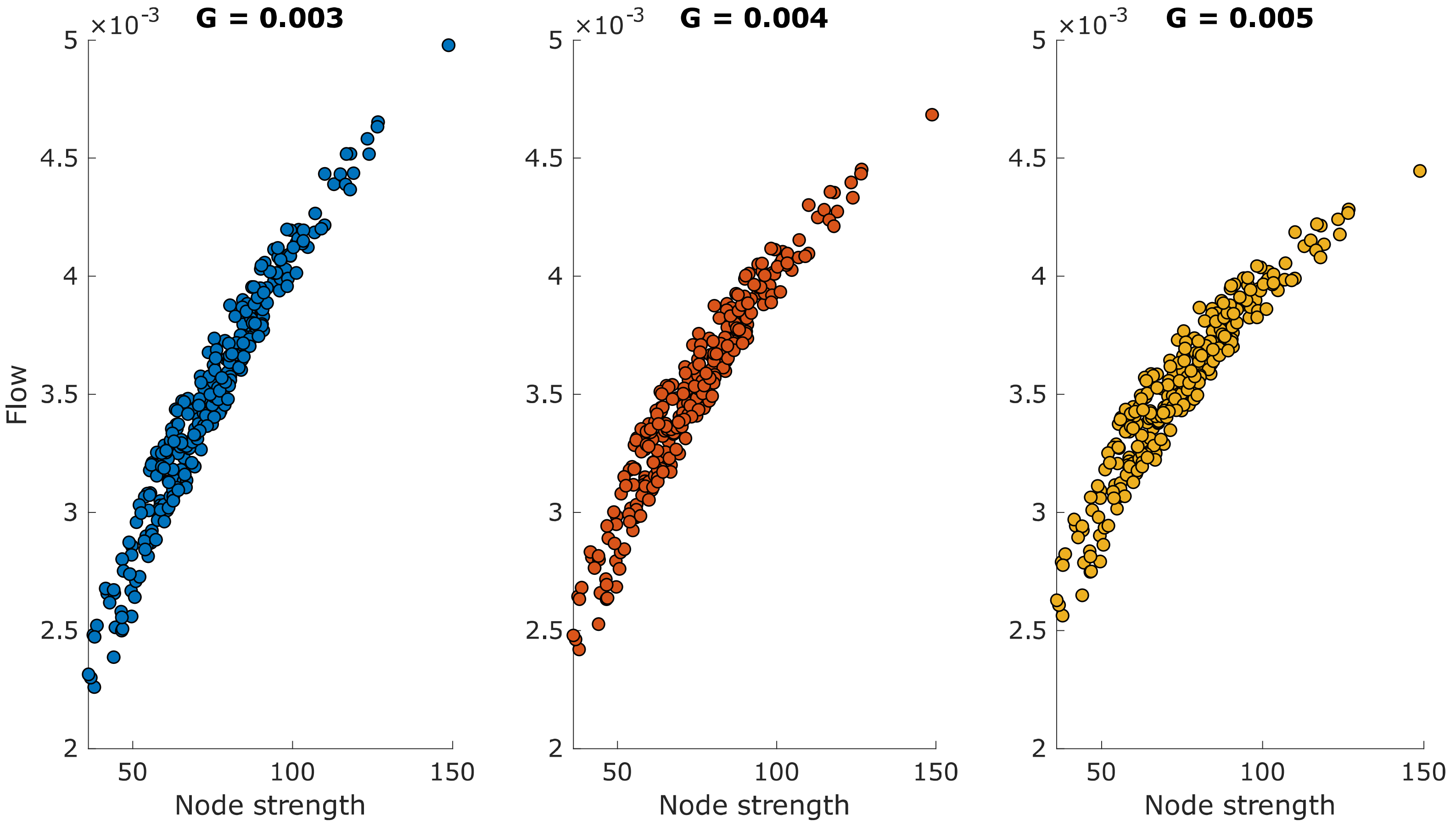

Figure S9. Node-level flow distribution for the MFM with linear firing rate on the MICA-MICs dataset

Flow through a node as a function of node strengths, for nodes following MFM dynamics with the non-linear firing rate function (9) replaced by a linear firing rate equation, evolving over the SC derived from the MICA-MICs dataset. This function was derived by fitting a line through the lower and upper bounds of the firing rate (*H*) corresponding to the minimum and maximum values of the input (*x*), which is first calculated by substituting all S_i with the lower and upper bounds of *S* (bounded between 0 and 1) respectively.

The values of the global scaling parameter for which the flow values are computed are the same as that used in the standard MFM (Fig.3), for comparison. The change in the firing rate equation clearly removes the dependence of the flow structure on *G* as is the case in Fig.3. Additionally, the flow structure of the "linear MFM" now conforms to one of the three flow structures in (Harush and Barzel, 2017) - Degree driven.
